# Screening of the Seed Region of *MIR184* in Keratoconus Patients from Saudi Arabia

**DOI:** 10.1155/2015/604508

**Published:** 2015-08-24

**Authors:** Khaled K. Abu-Amero, Inas Helwa, Abdulrahman Al-Muammar, Shelby Strickland, Michael A. Hauser, R. Rand Allingham, Yutao Liu

**Affiliations:** ^1^Department of Ophthalmology, College of Medicine, King Saud University, Riyadh, Saudi Arabia; ^2^Department of Cellular Biology and Anatomy, the Medical College of Georgia, Georgia Regents University, Augusta, GA, USA; ^3^Department of Medicine, Duke University Medical Center, Durham, NC, USA; ^4^Department of Ophthalmology, Duke University Medical Center, Durham, NC, USA

## Abstract

Micro-RNAs (miRNAs) are regulators of gene expression that control various biological processes. The role of many identified miRNAs is not yet resolved. Recent evidence suggests that miRNA mutations and/or misexpression may contribute to genetic disorders. Point mutations in the seed region of* MIR184* have been recently identified in Keratoconus (KC) patients with or without other corneal and lens abnormalities. We investigated mutations within* MIR184* in KC patients from Saudi Arabia and examined the relative expression of miR-184 and miR-205 in human cornea. Ethnically matched KC cases (*n* = 134) were recruited and sequencing was performed using PCR-based Sanger sequencing and analyzed using the Sequencher 5.2 software. Expression of miR-184 and miR-205 was profiled in postmortem unaffected ocular tissues obtained from donors with no history of ocular diseases. miR-184 expression was 15-fold higher than that of miR-205 in cornea samples. No mutation(s) within the screened genomic region of* MIR184* in KC cases was detected. This suggests that mutation in* MIR184* is a rare cause of KC alone and may be more relevant to cases of KC associated with other ocular abnormalities. The increased expression of miR-184 versus miR-205 in normal cornea samples implies a possible role of miR184 in cornea development and/or corneal diseases.

## 1. Introduction

Keratoconus (KC) is the most common primary ectatic disease of the cornea and one of the major indications for corneal transplant in the developed countries [1, 2]. KC is a lifelong condition that commonly occurs at puberty or during the second decade of life with an estimated prevalence of 1:500–2000 in the general population [3, 4]. It is usually associated with a significant impact on the patients' quality of life and represents a social and economic burden [5]. The severity of the disease varies considerably from cases that are mild or clinically asymptomatic at the early stage to severe progressive conical protrusion and vision impairment requiring corneal transplantation [1]. Accordingly, prognosis in KC is improved by early diagnosis and treatment [6].

Despite the intensive research, the etiology and pathogenesis of KC are poorly understood [7]. However, there is considerable evidence supporting the role of genetic predisposition to KC especially with increased incidence among families, ethnic groups, and twins [8–10]. Among the various reported KC-associated candidate genes are* VSX1* (visual system homeobox 1),* MIR184* (microRNA 184), and* DOCK9* (dedicator of cytokinesis 9) in addition to other candidate single nucleotide polymorphisms (SNPs) in other genetic loci [11–14]. Evidence regarding the contribution of these candidate genes remains controversial [2, 11, 12, 15–17].

MicroRNAs (miRNAs) are small (19–25 nucleotides), noncoding, and regulatory RNAs that bind to the 3′ untranslated region (UTR) of mRNA of target genes mediating mRNA degradation and suppression of translation [18]. Mutations in miRNA have previously been reported in association with various human diseases. In 2009, Mencía et al. [19] identified two mutations in the seed region of* MIR96* in two Spanish families affected by progressive hearing loss. Since then, the potential role of miRNA mutations in genetic diseases has been extensively investigated. Mutations in miRNA genomic regions have been identified in prostate and ovarian cancer [20, 21]. In this context, miRNAs have been regarded as a potential biomarker and a possible therapeutic target for a variety of disorders. There is a tissue and developmental stage-specific expression pattern for miRNA, which suggests a potentially important functional and developmental role in these tissues [22, 23].

Ocular tissues have a specific expression pattern of the different types of miRNAs [24]. The most abundantly expressed miRNA in the cornea and lens is miR-184 [22, 24]. In the cornea, the expression of miR-184 is cell type-specific. It is localized primarily in the basal and the immediate suprabasal cells of the corneal epithelium [24] and endothelium; however, it is not expressed in the limbus or conjunctival epithelia [22]. This spatial-specific expression further highlights the potential role of miR-184 in regulating cellular-specific functions in the eye.

Hughes et al. 2011 [14] have reported a heterozygous C-to-T transition (r.57c>T) within the* MIR184* seed region in a Northern Irish family in which 18 individuals from 3 generations were affected with KC associated with cataract. More recently other studies reported similar mutations in* MIR184*. In 2013, Lechner and colleagues [25] identified 2 heterozygous substitution mutations in the seed region of* MIR184* (+3A>G and +8C>A) in 2 patients with isolated KC. Similarly, another group [26] identified a c.57C>T mutation in* MIR184* affecting members of the same family. Individuals with c.57C>T mutation in* MIR184* display different corneal abnormalities including congenital cataract with keratoconus or corneal thinning but no keratoconus. miR-184 seems to play its biological role through the competitive inhibition of the binding of miR-205 to its mRNA target that encodes inositol polyphosphate-like 1 (INPPL1) and integrin, beta 4 (ITGB4) [14]. To further explore the role of* MIR184 *sequence variants in KC, we screened a group of KC patients from Saudi Arabia for mutations in the seed region of* MIR184. *Furthermore, taking into account the potential inhibitory effect of miR-184 on miR-205, we also examined the relative expression of miR-184 versus miR-205 in normal human cornea samples.

## 2. Materials and Methods

### 2.1. Study Population

This study adheres to the tenets of the Declaration of Helsinki and was approved by the ethical committee of College of Medicine, King Saud University (Riyadh, Saudi Arabia). All participants signed an informed consent. Study subjects were self-identified as Saudi Arabians. Furthermore, their ethnicity was confirmed through a database of Arab families of Saudi Arabian origin. Patients (*n* = 134; Table 1) were recruited from the anterior segment clinic at the Department of Ophthalmology after examination. Selection criteria for KC patients have been previously described [27–30]. In brief, KC cases were diagnosed using Schimpff flow-based corneal elevation mapping. KC Cases were defined as having posterior corneal elevation ≥ +20 *μ*m within the central 5 mm and inferior-superior dioptric asymmetry (*I*-*S* value) > 1.2 diopters (D), with the steepest keratometry > 47 D. Thorough family history was obtained for each patient. Sporadic cases were defined as those without family history of KC and their immediate family members were not affected. Exclusion criteria for KC cases were refusal to participate and post-LASIK ectasia as well as secondary causes for corneal disease including trauma, corneal surgery, Ehlers-Danlos syndrome, osteogenesis imperfecta, and pellucid marginal degeneration.

### 2.2. DNA Sequencing

**Figure 1:**
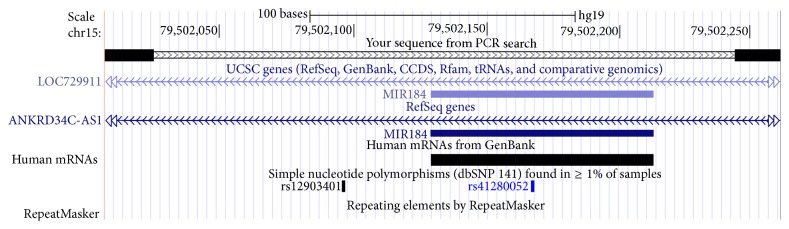
Chromosomal map showing the primers used in our study using UCSC genome browser. The figure shows that there are no common SNPs located in either the forward primer or reverse primer. Two common (rs12903401 and rs41280052) SNPs are located in the PCR amplicon.

DNA was isolated from peripheral blood samples as described previously [28, 29, 31]. Briefly, DNA was extracted from the buffy layer using the illustra blood genomicPrep Spin Kit (GE Healthcare Life Sciences, Buckinghamshire, UK). DNA quantity was measured using NanoDrop (Thermo Scientific, Wilmington, DE, USA). The genomic region containing* MIR184* was sequenced using PCR-based Sanger sequencing. Primers flanking the entire transcribed sequence of* has-mir-184* were designed using Primer3 software [32, 33]. For the PCR, the primer sequences and amplicon genetic locus were verified using GRCh37/hg19 in UCSC human genome browser (chr15:79502007–79502261) (Table 2). Based on the UCSC* in silico* PCR search (https://genome.ucsc.edu/cgi-bin/hgPcr), no common variants were localized to the two primer sequences (Figure 1).

PCR was performed using Platinum Taq DNA polymerase from Life Technologies (Grand Island, NY, USA) with the addition of 5 M betaine from Sigma-Aldrich (St. Louis, MO, USA). 10 ng genomic DNA was used in each PCR reaction. The PCR amplifications were performed using Eppendorf Mastercycler PCR machines with a “touchdown” strategy whereby the annealing temperature is lowered incrementally over the course of the reaction. Initial thermocycler conditions were as follows: 94°C for 30 s, 65°C for 30 s, and 72°C for 30 s. After two cycles using an annealing temperature of 65°C, this step was lowered to 63°C for two cycles and then it was lowered to 61°C for two cycles, 59°C for two cycles, 57°C for two cycles, and finally 55°C for 30 additional cycles (40 cycles total). Additional extension step was added at the end with 72°C for 10 minutes. Completed PCR reactions were purified and sequenced in the forward direction using BigDye chemistry (Applied Biosystems, Carlsbad, CA). Potential mutations were confirmed by additional sequencing in the reverse direction. All the sequences were analyzed using Sequencher 5.2 software package (Gene Codes, Ann Arbor, MI).

### 2.3. miRNA Sequencing

Ocular tissues including cornea, ciliary body, retina, and trabecular meshwork were obtained from postmortem eyes donated by four healthy individuals with no history of ocular diseases within 24 hours of death. After dissection, ocular tissues were placed into RNAlater overnight at 4°C after which RNAlater was removed and samples were stored at −80°C. Total RNA was isolated from 2 cornea samples, 2 ciliary body samples, 2 retina samples, and 4 trabecular meshwork samples using mirVana miRNA isolation kit from Life Technologies according to the recommended protocols from the manufacturer. The quality of isolated RNA was assessed on a 1% agarose gel based on the relative abundance of 18S and 28S subunits of ribosomal RNA. The concentration of the total isolated RNA including miRNAs was measured using a single channel NanoDrop 2000 (Nanodrop, Wilmington, DE, USA).

Small RNA sequencing library was generated using TruSeq Small RNA Sample Preparation Kit from Illumina (San Diego, CA, USA) according to the recommended protocol from the manufacturer. Briefly, 1 *μ*g of total RNA including miRNA was ligated with an RNA 3′ adapter and an RNA 5′ adapter. Those small RNAs ligated with both 3′ and 5′ adapters were reverse-transcribed followed by PCR to create cDNA constructs with the integration of sample-specific index. The amplified cDNA constructs were gel-purified to enrich amplified miRNAs and validated with Agilent Bioanalyzer 2100 using High Sensitivity DNA chips. The validated small RNA sequencing libraries were normalized, denatured, and loaded to Illumina MiSeq Personal Sequencing System using MiSeq reagent kit v2 with 50 cycles. Sequencing reads for all 10 samples generated in MiSeq were analyzed using MiSeq small RNA data analysis pipeline as previously described [34]. The trimmed sequences were aligned against miRbase for both miRNA and its loop sequences. The expression levels of miR-184 and miR-205 were examined in all 10 ocular samples. The expression level for each miRNA was normalized as the number of sequence reads per million of total sequencing reads for each tissue.

## 3. Results

**Figure 2:**
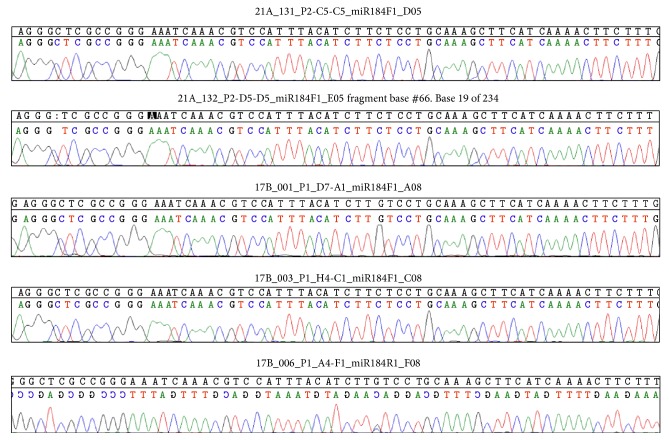
Representative sequence chromatograms showing no substitution mutations in five KC patients from the Saudi Arabian group. Sequences were compared to the reference coding sequence* MIR184*.

Our study included 134 KC cases from Saudi Arabia. The genomic region covering the* has-mir-184* precursor was successfully amplified with PCR with one unique product at a size of 255 bp. PCR products were successfully sequenced for all KC patients. The sequencing data was examined in reference to the sequence of has-mir-184 using Sequencher software (Gene Codes Corporation, Ann Arbor, MI) (Figure 2). No point mutations, small insertions, or small deletions were identified in the precursor region of miR-184 in any of the 134 KC patients.

**Figure 3:**
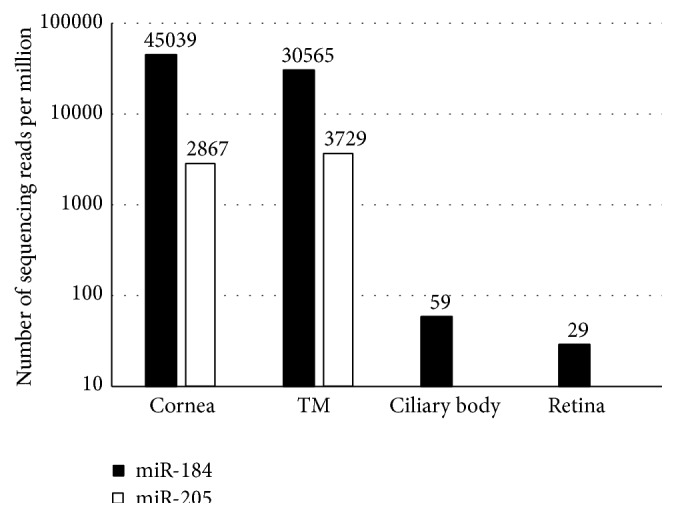
The relative expression level of miR-184 and miR-205 in different human ocular tissues (cornea, trabecular meshwork (TM), ciliary body, and retina) using miRNA sequencing with Illumina MiSeq Personal Sequencing System. (*Y*-axis logarithmic scale of 10).

Our microRNA sequencing data in postmortem unaffected human ocular samples with Illumina MiSeq identifies the expression of more than 340 mature miRNAs, including both miR-184 and its competitor miR-205 (Figure 3). Our data indicates that miR-184 is the most abundantly expressed miRNA in the cornea with a mean of 45,039 reads per million sequencing reads and it is also highly expressed in the trabecular meshwork (30,565 reads per million sequencing reads). However, the expression of miR-184 is low in the ciliary body (59 reads per million sequencing reads) and retina (29 reads per million sequencing reads). On the other hand, we indicate that miR-205 is also highly expressed in the cornea and the trabecular meshwork with a mean of 2867 and 3729 reads per million sequencing reads, respectively. However, similar to the miR-184 data, miR-205 has a negligible expression in both the ciliary body and the retina. The expression of miR-184 is almost 15-fold and 8-fold higher than that of miR-205 in the human cornea and the trabecular meshwork samples, respectively.

## 4. Discussion

miRNAs are regulators of gene expression that modulate various biological functions including proliferation, apoptosis, and differentiation [18]. Most ocular miRNAs are expressed in a tissue-specific pattern. miR-184 is most abundantly expressed in the cornea and lens epithelia. More notably, its expression is restricted to the corneal basal and immediate suprabasal layers [24]. It has also been previously reported that the expression of miR-184 in the cornea was downregulated in the reepithelializing cells of wounded corneal epithelium. However, increased corneal epithelial proliferation had no effect on the expression of miR-184. These observations suggest that the expression of miR-184 is independent of the proliferative stage of corneal epithelia but may be delineating their differentiated phenotype. In contrast, the expression of miR-184 in the lens was more significantly detected in the germinative layer of epithelial cells and was more uniformly distributed at an earlier developmental stage [24]. This restricted expression pattern in the lens and cornea suggests a unique role of miR-184 in these particular ocular structures and may be specifically related to the regulation of proliferation versus differentiation of cornea and lens epithelia. In accordance with other reports [24, 35], our miRNA expression data indicated miR-184 as the most abundantly expressed miRNAs in postmortem human cornea samples obtained from donors with no history of ocular diseases. These expression data support the recent publication by Teng et al. 2015 [36] reporting abundance of miR-184 in postmortem human cornea samples. Our findings together with that by Teng and colleagues [37] are the first to report the abundant expression of miR-184 in normal human cornea.

Despite the available data regarding the distinct expression pattern and level of miR-184 in ocular tissues, little is known about the biological function of this miRNA in these two tissues. This intriguing biological role has been primarily investigated through cotransfection of Hela cells with miR-184 and miR-205 and using miR-184 and miR-205 antagonists in primary human epidermal keratinocytes [39]. Accordingly, it was reported that miR-184 prevents knockdown of INPPL1 and ITGB4 by miR-205 and rescued their levels in cell culture [14, 39]. INPPL1 and ITGB4 are suggested to regulate corneal healing, a role that seems consistent with the pathogenesis of KC and its association with mutations in* MIR184 *[14]. The potential role of miR-184 as a competitive inhibitor of miR-205 was further confirmed by Hughes and colleagues [14]. These functional findings further suggest* MIR184 *mutation as a potential candidate for various ocular defects especially those related to the cornea and lens.

In 2009, Mencía et al. [19] reported the first implication of miRNA mutation in a nonsyndromic progressive hearing loss disorder affecting a Spanish family. In a similar context, various reports have subsequently highlighted an association between point mutations within the seed region of* MIR184* and KC with/without other ocular abnormalities including cataract and myopia (Table 3) [14, 25, 40–42]. Only one of these studies reported mutation in the seed region of* MIR184* affecting patients with KC which was not associated with cataract [25]. In that study, a substitution mutation within* MIR184* was present in 2 sporadic KC cases. Moreover, only 1 of these 2 KC patients had KC without cataract. However, these 2 cases altogether accounted for 0.25% of the screened cases (2 of 780 KC-affected patients). On the other hand, no sequence variants were identified in either the myopia subjects, control subjects, or the rest of the sporadic KC subjects [25]. This indicates that this sequence variant may account for a very small percentage of KC cases and may be more relevant to the occurrence of KC in accordance with other ocular defects (as cataract). However, in this report, according to dbSNP 141, a common SNP, rs58249183 (minor allele frequency ≥ 1%), is localized in one of the PCR primers. If we assume that this patient was heterozygous with SNP rs58249183, PCR with primers overlapping this SNP would only amplify one of the two DNA strands. Thus, the information on the other strand would be missed raising a remote possibility of some false negatives with PCR-based DNA sequencing. On the other hand, all the other reports detected this mutation in patients affected with sporadic KC associated with cataract or cataract with corneal thinning but no KC [14, 26].

Likewise, substitution mutation within the seed region of* MIR184 *has also been mapped in patients with endothelial dystrophy, iris hypoplasia, congenital cataract, and stromal thinning, not associated with keratoconus (EDICT syndrome) [38, 43] as well as familial KC with cataract [14]. Moreover, this later report by Hughes et al. [14] did not provide adequate description of the iris and lens phenotype. Thus, they might have missed some of the other ocular phenotypic features that may have been associated with this mutation [44]. We recommend further studies with more detailed description of the phenotype associated with this mutation to accurately characterize the phenotype related to this genetic variant.

Taken together, the lack of sequence variants within the seed region of* MIR184* in our group of KC patients from Saudi Arabia is still consistent with the rarity of the occurrence of this mutation in patients with KC only.

## 5. Conclusion

Our report represents a follow-up study on the association between* MIR184* mutations and KC. Based on our findings and in accordance with previous reports, variants within* MIR184* seem to play a limited role in the susceptibility for KC alone. However,* MIR184* may be an attractive genetic candidate in implication with the cooccurrence of KC with other corneal abnormalities such as cataract. Our data regarding the abundant expression of miR-184 as compared to miR-205 in normal human cornea samples further highlight the potential role of miR-184 in the cornea. However, absence of* MIR184* sequence variant in this population of KC patients without any other concomitant ocular abnormalities is consistent with previously published reports. The absence of sequence variants in patients with KC only suggests that* MIR184 *may have a more pronounced role as a candidate gene for other corneal/lens abnormalities with or without KC. This can be further confirmed by examining the relative expression of miR-184 and miR-205 in KC-affected cornea samples. Comparing the expression of miR-184 and miR-205 in control versus KC cornea samples as well as corneas of other ocular abnormalities will reveal the contribution of miR-184 and miR-205 to KC and/or other ocular defects. In summary, our findings recommend more elaborative genetic and functional studies to elucidate the expression/role of miR184 in the normal versus diseased cornea.

## Figures and Tables

**Table 1:** The study population.

Category	Number of subjects	Families included
Familial cases	72	48
Sporadic cases (isolated)	62	0
Total	134	48

**Table 2:** The primer used for *MIR184* sequencing.

Primer	Primer sequence (5′-3′)	Product size (bp)	*T*_*m*_
*has-mir-184*	F: CAGAGGGGCTTTGAATTTGA	255	62.5°C
	R: CCCATCACGCAAGTGCAG		60.2°C

Note: F: forward primer; R: reverse primer; bp: base pair; *T*_*m*_: melting temperature.

**Table 3:** Summary of miR-184 mutations in keratoconus patients from different studies.

Study	Population	Number of cases	Phenotype	Mutation within *MIR184*	Mutation prevalence
Iliff et al. [38]	Nonspecified	8 (same family)	Anterior segment dysgenesis, corneal endothelial dystrophy, iris hypoplasia, congenital cataract, corneal stromal thinning (EDCIT syndrome)	+57C>T	In all cases (8) None in controls (2)

Hughes et al. [14]	Northern Irish	18 (same family)	Severe anterior KC and early onset anterior polar cataract	+57C>T	Only in affected single and pooled samples

Bykhovskaya et al. [26]	Spanish (Galicia, Spain)	5 (same family)	Congenital cataract with corneal thinning, congenital cataract with keratoconus (proband)	+57C>T	3 affected individuals

Lechner et al. [25]	Mixed ethnicity (European Caucasian-South Indian)	780 (unrelated)	KC and no lens or iris abnormalities KC with cataract but no iris abnormalities	+3A>G +8C>A	2 affected individuals (0.25% of screened cases)

Current study	Middle Eastern (Saudi Arabian)	134 familial and sporadic	Keratoconus without congenital cataract	N/A	0
